# Appetitive traits and relationships with BMI in adults: Development of the Adult Eating Behaviour Questionnaire

**DOI:** 10.1016/j.appet.2016.05.024

**Published:** 2016-10-01

**Authors:** Claudia Hunot, Alison Fildes, Helen Croker, Clare H. Llewellyn, Jane Wardle, Rebecca J. Beeken

**Affiliations:** Health Behaviour Research Centre, Department of Epidemiology and Public Health, University College London, London WC1E 6BT, United Kingdom

**Keywords:** Appetitive traits, Appetite, Adults, Overweight, Obesity, Food responsiveness, Satiety responsiveness, Questionnaire

## Abstract

The Child Eating Behaviour Questionnaire (CEBQ) is a validated parent-report measure of appetitive traits associated with weight in childhood. There is currently no matched measure for use in adults. The aim of this study was to adapt the CEBQ into a self-report Adult Eating Behaviour Questionnaire (AEBQ) to explore whether the associations between appetitive traits and BMI observed in children are present in adults. Two adult samples were recruited one year apart from an online survey panel in 2013 (n = 708) and 2014 (n = 954). Both samples completed the AEBQ and self-reported their weight and height. Principal component analysis (PCA) was used to derive 35 items for the AEBQ in Sample 1 and confirmatory factor analysis (CFA) was used to replicate the factor structure in Sample 2. Reliability of the AEBQ was assessed using Cronbach’s α and a two week test-retest in a sub-sample of 93 participants. Correlations between appetitive traits measured by the AEBQ and BMI were calculated. PCA and CFA results showed the AEBQ to be a reliable questionnaire (Cronbach’s α > 0.70) measuring 8 appetitive traits similar to the CEBQ [Hunger (H), Food Responsiveness (FR), Emotional Over-Eating (EOE), Enjoyment of Food (EF), Satiety Responsiveness (SR), Emotional Under-eating (EUE), Food Fussiness (FF) and Slowness in Eating (SE)]. Associations with BMI showed FR, EF (p < 0.05) and EOE (p < 0.01) were positively associated and SR, EUE and SE (p < 0.01) were negatively associated. Overall, the AEBQ appears to be a reliable measure of appetitive traits in adults which translates well from the validated child measure. Adults with a higher BMI had higher scores for ‘food approach’ traits (FR, EOE and EF) and lower scores for ‘food avoidance’ traits (SR, EUE and SE).

## 1 Introduction

The current obesity epidemic has reached widespread proportions, showing a combined increase in adult overweight and obesity prevalence of 27.5% worldwide between 1980 and 2013 (Ng et al., 2014). This is particularly concerning given that obesity is a major contributor to a number of physical and psychological health conditions, as well as increases in mortality (Flegal, Kit, & Orpana, 2013). There is therefore a desire to understand the mechanisms behind the aetiology of obesity.

At an individual level, factors such as food overconsumption and decreases in physical activity are interacting to determine weight gain (Llewellyn & Wardle, 2015). These behaviours are thought to be influenced both by the environment and by genetically determined appetitive traits (Llewellyn, van Jaarsveld, Plomin, Fisher, & Wardle, 2012), defined as a set of stable predispositions towards food (Carnell, Benson, Pryor, & Driggin, 2013). The behavioural susceptibility theory of obesity (Llewellyn & Wardle, 2015) suggests that individual differences in these traits relate to susceptibility to gain weight (or not) in response to the current obesogenic environment.

The majority of studies exploring appetitive traits and weight have used validated and reliable questionnaires, which removes the costly obstacles of laboratory and neural measurements of appetite and makes data available for large populations (Carnell & Wardle, 2007). A considerable number of questionnaires have been used to measure appetite. The most widely used adult tools are the ‘Three Factor Eating Questionnaire’ (TFEQ) (Stunkard & Messick, 1985) and the ‘Dutch Eating Behaviour Questionnaire’ (DEBQ) (Van Strein, Frijters, Bergers & Defares, 1986); and with children, the Child Eating Behaviour Questionnaire (CEBQ) (Wardle, Guthrie, Sanderson, & Rapoport, 2001). These 3 questionnaires have improved understanding of the individual appetitive traits that increase a person’s risk of weight gain or resistance to weight loss. However, although important in their own right, neither the TFEQ nor the DEBQ capture all of the specific traits measured by the CEBQ, a parent-report questionnaire validated for use with children between the ages of 3 and 13 years.

The CEBQ measures 8 appetitive traits; 4 ‘food approach’ traits; Food Responsiveness (FR), Emotional Over-Eating (EOE) and Enjoyment of Food (EF), Desire to Drink (DTD) and 4 ‘food avoidance’ traits; Satiety Responsiveness (SR), Emotional Under-Eating (EUE), Food Fussiness (FF) and Slowness in Eating (SR). In contrast, the DEBQ captures 3 aspects of eating (Emotional Eating, Restraint and External Eating) and the TFEQ captures Cognitive Restraint, Disinhibition and Hunger. Although the DEBQ does include an emotional eating scale, unlike the CEBQ, it does not contain separate sub-scales for emotional over-eating and emotional under-eating. Similarly the Disinhibition scale of the TFEQ incorporates items relating to both emotional eating and food responsiveness, rather than treating them as distinct constructs.

Numerous studies have demonstrated that the ‘food approach’ traits captured by the CEBQ are positively correlated with weight in childhood (Croker et al., 2011, Santos et al., 2011, Sleddens et al., 2008) whereas, children who score highly on ‘food avoidance’ traits are less susceptible to overeating, and have lower weight (Fuemmeler et al., 2013, Spence et al., 2011, Webber et al., 2009). Longitudinal studies using the CEBQ also provide support for the hypothesis that these appetitive traits contribute to weight gain rather than the other way around (Van Jaarsveld, Llewellyn, Johnson, & Wardle, 2011).

There is some evidence from studies using the CEBQ that appetitive traits vary with age (Ashcroft, Semmler, Carnell, van Jaarsveld, & Wardle, 2008). However, studies exploring changes in appetitive traits across the life course have been limited by the lack of a matched self-report measure of these specific appetitive traits for adults. Food Responsiveness and Satiety Responsiveness have particularly strong relationships with weight in children, but neither is adequately captured by existing measures of appetite in adulthood. Measurement of these traits in adults would contribute to our understanding of how these specific traits influence weight gain at older ages (French, Epstein, Jeffery, Blundell, & Wardle, 2012).

There is already a Baby Eating Behaviour Questionnaire (BEBQ) (Llewellyn, van Jaarsveld, Johnson, Carnell, & Wardle, 2011) that enables the measurement of the traits assessed by the CEBQ in infants. The addition of an Adult Eating Behaviour Questionnaire (AEBQ) would provide the field with three life-stage appropriate measurement instruments that assess the same eating behaviours. This would make it possible to longitudinally track appetitive traits from infancy (BEBQ) and childhood (CEBQ) into adulthood (AEBQ), to give a better picture of the association between appetitive traits and weight across the life-course. Scores on an AEBQ could also be used to inform interventions to help individuals to control their weight, by providing tailored feedback on managing appetitive trait responses. It may also enable identification of individuals at risk of weight gain to inform targeted obesity prevention efforts.

The aim of this study was therefore to adapt the parent-report ‘Children’s Eating Behaviour Questionnaire’ into a psychometrically valid self-report ‘Adult Eating Behaviour Questionnaire’, and to explore whether relationships between appetitive traits and BMI observed in children can also be seen in an adult sample.

## 2 Methods

### 2.1 Development of the Adult Eating Behaviour Questionnaire

***Generation of items***: The 35 items from the CEBQ were changed from the “My child …” format to a self-complete “I ...” format (e.g. “My child loves food” was changed to “I love food”) and the original response options (‘never’, ‘rarely’, ‘sometimes’, ‘often’ and ‘always’) were retained. Ten researchers working in the area of Energy Balance completed the self-report version of the CEBQ and discussed their experiences. The researchers described how the Desire to Drink scale was difficult to complete. Items from the CEBQ such as “My child is always asking for a drink” had been adapted to “I am always asking for a drink” for the AEBQ and it became unclear what type of drink (i.e. alcoholic versus non-alcoholic) was being referred to. Additionally, the item “My child is always asking for food” from the FR construct in the CEBQ, which became “I am always asking for food” in the AEBQ, was difficult for adults to relate to. It was therefore agreed that the 3 items from the Desire to Drink scale, and the “I am always asking for food item” from the FR scale should be eliminated.

Further refinement of the questionnaire took place in 3 group discussions with a panel of clinical psychologists, behavioural scientists, dieticians, and authors of the original CEBQ. The panel initially reviewed the remaining items from the original CEBQ for any obvious gaps or additional problem areas. It was suggested that a measure of hunger experience (H), which could not be captured by the CEBQ because parents are unable to accurately determine their child’s experienced level of hunger, should be added (Wardle et al., 2013). It was also agreed that aspects of Food Responsiveness that related to food cues a parent would not have been able to comment on should also be included. Following this discussion, potential items for the Hunger scale were identified for review, and additional items for the Food Responsiveness scale were developed by the authors for piloting. Finally the panel reviewed all included and excluded items to ensure no further additions/removals were felt to be required. A group consensus was reached and the total number of items following these additions, and the removal of the Desire to Drink scale, was 49.

***Piloting*.** The extended version of the AEBQ was piloted online in an opportunity sample of 49 adults (21–73 years old), 36 women (79.6%) and 13 men (20.4%). Colleagues at University College London were asked to circulate a link to the questionnaire to their friends and family from a range of professional backgrounds. Participants were invited to comment on each individual item and on the questionnaire as a whole. Piloting led to changes in the response options from ‘never’, ‘rarely’, ‘sometimes’, ‘often’ and ‘always’, to ‘strongly disagree’, ‘disagree’, ‘neither agree not disagree’, ‘agree’ and ‘strongly agree’ because participants commented that the original response options did not fit with the questions. The new response options were tested with a small convenience sample (two females and three males, aged 31 ± 7 years). This answer format appeared to be more meaningful and better understood by this sample.

Piloting also led to the deletion of the item “Given the choice, I would always have food in my mouth” because several participants commented that it “*sounded a bit odd*” or was “*over the top*”. A second item (“*I am interested in food*”) was eliminated because participants reported they found the meaning ambiguous. The remaining 47 item version of the AEBQ was included in the Principal Component Analysis (PCA).

### 2.2 Assessing the factor structure of the Adult Eating Behaviour Questionnaire

***Samples***. Two samples of adults aged 18 or over [Sample 1 (2013) and Sample 2 (2014)] were recruited one year apart, from an online provider of sampling and data collection for survey research (Research Now). Research Now holds a panel of over 200,000 UK residents who have consented to answer online questionnaires. Ethical approval was granted by University College London Research Ethics Committee.

***Measures***. Participants provided demographic information on ethnicity, level of education, working status, and income, in addition to completing the 47 item AEBQ. Self-reported weight and height measures were obtained for BMI calculation.

***Analysis***. PCA was used to uncover the underlying structure of the AEBQ from Sample 1 responses (Field, 2013) using SPSS version 22.0. All AEBQ items showed high inter-correlation greater than 0.3, but no multi-collinearity was observed. Data were extracted using oblimin rotation to allow for correlation between components and eigenvalues above 1 were retained. There were no missing cases for the scales. An iterative process was used to gradually remove items that had unacceptable factor loadings (<0.3), or that loaded highly onto two components. Items with factor loadings above 0.3 were retained because these are considered statistically meaningful with a large sample size (Field, 2013).

Confirmatory factor analysis (CFA) was carried out on responses from Sample 2 by specifying the model obtained from the PCA in Sample 1 (Model 1), using SPSS AMOS version 22.0. We looked at modification indices and covariances of error terms on the same factors (component in PCA) to test different models. Model fit was assessed using the following indices: root mean-square error of approximation (RMSEA); chi-square test (χ^2^); normed fit index (NFI) and comparative fit index (CFI). Smaller values for RMSEA (ideally ≤0.06) and values approaching 0.90 for NFI and CFI (ideally >0.90) are indicative of good model fit (Hu & Bentler, 1999). The Chi-square test is a measure of the difference between observed and expected covariance matrices and should be non-significant. However, the Chi-square test readily reaches significance with large sample sizes even when all other indices indicate a good fit (Dugard, Todman, & Staines, 2010). Lower AIC (Akaike’s Information Criteria) and BIC (Bayesian Information Criterion) values were used to select the best fitting model (Dugard et al., 2010). Cronbach’s alphas were also calculated for each of the scales obtained from the best model fit to assess the internal consistency of the AEBQ. Cronbach’s alphas greater than 0.70 were accepted as a good measure of consistency for each appetitive trait (Field, 2013). Individual mean scores were calculated for each of the AEBQ scales obtained after CFA on the whole Sample 2.

Test re-test reliability of the AEBQ was assessed by repeating it in a sub-sample of 93 participants [19 males (20.4%) and 74 females (79.6%)] two weeks after initial completion. The sub-sample were selected at random from Sample 2 by *Research Now* and invited to complete the online questionnaire a second time. The test re-test reliability of the measure was assessed using Intra-Class Correlation coefficients (ICC) (McGraw & Fleiss, 1996) and Cronbach’s alpha measurements based on the average inter-item correlations (Field, 2013).

Correlations between scales and BMI in Sample 2 were determined using Pearson’s correlation coefficient for normally distributed scales and Spearman’s rho for non-normally distributed scales.

## 3 Results

The AEBQ was initially completed by 708 adults aged 18–81 years (Sample 1; see Table 1). Sample 1 had a mean age of 39 ± 17, with a mean BMI of 26.10 ± 5.81. One year later the AEBQ was completed by a second sample of 954 adults aged between 18 and 79 (Sample 2; see Table 1). The mean age of Sample 2 was 44 ± 13 and the mean BMI was 26.07 ± 5.80. A sub-sample of 93 participants from Sample 2 (mean age 49 ± 13; see Table 1) completed the AEBQ a second time two weeks later to assess test-retest reliability. Both samples were mostly white (Sample 1: 90.3%, Sample 2: 90.5%), and the majority of participants were female (Sample 1: 52.5%, Sample 2: 57.3%) (see Table 1).

### 3.1 Principal Component Analysis

PCA revealed a 35 item questionnaire which loaded onto 7 components. Twelve items from the original 47 were excluded through the iterative process. Each remaining item had the highest loading on to a single component [except for ‘I often feel hungry when I am with someone who is eating’ which loaded onto the Food Responsiveness construct and also Enjoyment of Food (0.31) (Table 2)], and explained the highest variance. The 7 components encompassed three ‘food approach’ scales and four ‘food avoidance’ scales. The ‘food approach’ scales were: Hunger and Food Responsiveness (which loaded onto a single component), Emotional Over-Eating, and Enjoyment of Food. The four ‘food avoidance’ scales were: Satiety Responsiveness, Emotional Under-Eating, Food Fussiness and Slowness in Eating. These 7 components had an average communality of 0.64 and explained 64.27% of the variance. The final model fit the data well and resembled the CEBQ (Table 2) (Appendix 1).

### 3.2 Confirmatory factor analysis

The 35 AEBQ items were entered into a 7 factor CFA, where indicators (items) of each factor (component) loaded onto their own factor, based on the PCA results (Model 1). All factor loadings were >0.3; Hunger and Food Responsiveness together (0.39–0.76), Emotional Over-Eating (0.70–0.88), Enjoyment of Food (0.72–0.89), Satiety Responsiveness (0.57–0.83), Food Fussiness (0.71–0.89) and Slowness in Eating (0.71–0.90). This resulted in a relatively decent model fit (RMSEA = 0.06, NFI = 0.87, CFI = 0.90, χ^2^(df = 539) = 2431.35, p < 0.001) (Hu & Bentler, 1999). After examining the modification indices and the co-varied error terms with the largest parameter changes that were part of the same factor, unexplained correlations relating to the combined Hunger and Food Responsiveness factor were identified (Dugard et al., 2010). Therefore Hunger and Food Responsiveness were split into two separate factors and each indicator was allowed to only load on to their respective factor (factor loadings ranged from 0.44 (*If my meals are delayed I get light-headed*) to 0.79 (*I often feel hungry*) for Hunger and from 0.55 (*When I see or smell food that I like*, *it makes me want to eat*) to 0.72 (*Given the choice*, *I would eat most of the time*) for Food Responsiveness). The 8 factor model (Model 2) produced a better model fit (RMSEA = 0.06, CFI = 0.91, χ^2^(df = 532) = 2254.66, p < 0.001) and lower AIC and BIC values (Table 3).

Cronbach’s alphas for each of the 8 scales were greater than 0.70 (range 0.75–0.90) indicating good internal reliability. Measures of test-retest reliability in the subsample of 93 participants also revealed good external reliability with all Cronbach’s alpha values greater than 0.70 (α: 0.73–0.91) (Field, 2013) (Table 4). Overall, the scales correlated with each other in the expected direction; ‘food approach’ scales were positively correlated with each other and negatively correlated with ‘food avoidance’ scales, and ‘food avoidance’ scales were positively correlated with each other (Table 5).

### 3.3 Associations with BMI

All of the scale values were normally distributed except for Enjoyment of Food which was skewed to the right. Results are presented for participants with BMIs greater than 14 and lower than 50, as this range was felt to reflect realistic values. Results showed small positive correlations between BMI and the ‘food approach’ scales; Food Responsiveness, Emotional Over-Eating and Enjoyment of Food; participants with higher BMI values scored higher on these scales. Small negative correlations were observed between BMI and the ‘food avoidance’ scales; Satiety Responsiveness, Emotional Under-Eating and Slowness in Eating. No relationships were found between BMI and either Hunger or Food Fussiness (Table 6).

## 4 Discussion

This is the first study to measure appetitive traits using the AEBQ, a new reliable self-report measure of appetitive traits in adulthood. Correlations between appetitive traits and BMI showed that adults with higher BMIs scored higher for Food Responsiveness, Emotional Over-Eating and Enjoyment of Food and lower for Satiety Responsiveness, Emotional Under-Eating and Slowness in Eating.

The AEBQ differs from the CEBQ in several ways including the addition of a Hunger scale and the removal of the Desire to Drink scale. However because the CEBQ is used as a set of subscales rather than as a single scale, this should not impact the ability to use the AEBQ in longitudinal studies with both the CEBQ and the BEBQ. Although such studies will not be able to measure Desire to Drink in adults, nor Hunger in children, the 7 remaining scales are the same across the two questionnaires. Importantly those traits found to have the strongest relationships with weight in childhood (Food Responsiveness and Satiety Responsiveness) are retained in the AEBQ.

The Desire to Drink scale was eliminated after piloting as it was deemed unsuitable for adult samples. Previous studies have reported no relationship between the CEBQ Desire to Drink scale and weight in children aged 3–13 (Santos et al., 2011, Sleddens et al., 2008, Sweetman et al., 2008, Viana et al., 2008), or in Malaysian adolescents who completed a self-report version of the CEBQ (Loh, Moy, Zaharan, & Mohamed, 2013). Therefore, the exclusion of this scale from the AEBQ is unlikely to be of significance for studies seeking to explore the association between appetitive traits and weight in older samples.

Hunger may be seen as an important aspect of appetite that was omitted from the CEBQ because of the parent-report nature of the questionnaire. However, while a Hunger scale was added to the AEBQ because adults can report their own experienced levels of hunger, this scale was not associated with weight. The new Hunger scale is a measure of physical hunger (e.g. stomach rumbles) unrelated to emotional or restraining situations as measured in the revised and shortened TFEQ (TFEQ- R18) (Karlsson et al., 2000, Stunkard and Messick, 1985). It is possible that people find it difficult to assess their level of physical hunger, perhaps due to its relationship to forms of disinhibition and issues with eating regulation (Karlsson et al., 2000). It is also likely that individuals differ in their perception and interpretation of what hunger actually means (Wardle, 1987). As seen in the factor loadings and the correlations between the scales, the relationship between Hunger and Food Responsiveness was strong, although the CFA ultimately revealed separating these scales provided the best model fit. While Hunger and Food Responsiveness appear to be overlapping constructs, substantial literature exists which distinguishes them as separate dimensions of eating (Meyer and Pudel, 1972, Schachter and Gross, 1968, Schachter, 1968, Stunkard and Fox, 1971). Future studies using the AEBQ should consider whether it is important to retain the Hunger scale as an important appetitive trait in adults.

The eight AEBQ scales were found to have good internal reliability (α: 0.762 to 0.881) (Field, 2013), in line with the CEBQ (Ashcroft et al., 2008) and the infant version, the BEBQ (Llewellyn et al., 2011). The AEBQ scales also showed good test-retest reliability (ICCs: 0.732 to 0.910), comparable to most CEBQ scales with the exception of Emotional Over-Eating and Emotional Under-Eating (Wardle et al., 2001), which appear to be more stable in adults. As with the CEBQ and BEBQ (Llewellyn et al., 2011, Wardle et al., 2001), positive correlations were observed between the four ‘food approach’ scales (H, FR, EOE and EF), and between the four ‘food avoidance’ scales (SE, EUE, SE and FF only with SR) of the AEBQ, while generally negative correlations were observed between the different types of scales.

The relationships between appetitive traits and BMI observed in this adult sample are similar to findings from the child literature where adiposity is consistently positively associated with ‘food approach’ scales and negatively associated with ‘food avoidance’ scales of the CEBQ (Mallan et al., 2013, Santos et al., 2011, Sleddens et al., 2008, Viana et al., 2008, Webber et al., 2009). Although the direction of associations in this study replicates those from studies in children using the CEBQ, they were more modest in our sample of adults. This may be indicative of appetitive traits exerting a differential influence on weight across the life course. Furthermore, adults may actively restrict their energy intake in an attempt to control their weight, which could suppress the impact of certain traits on BMI, whereas children typically do not exert such control over their eating. Future studies should examine the relationship between the AEBQ and measures of dieting or restrictive eating behaviours.

No significant association was found between BMI and the AEBQ Food Fussiness scale. It is possible that Food Fussiness in adults is directed towards a much smaller number of foods, while greater variation exists in relation to children’s Food Fussiness (Croker et al., 2011, Spence et al., 2011, Webber et al., 2009). Picky eating in adults is also associated with forms of unhealthy eating that may lead to higher BMIs in some cases (Kauer, Pelchat, Rozin, & Zickgraf, 2015). However relationships between Food Fussiness and BMI in children have also not always been consistent (Santos et al., 2011, Svensson et al., 2011).

There are limitations to this study. The adaptation of the AEBQ may have benefited from a more structured process of cognitive testing to assess its face validity (Banna, Vera Becerra, Kaiser, & Townsend, 2010). We received input from experts in the area, and piloted the questionnaire in an age-appropriate sample to refine the original items from the CEBQ that were initially just translated into self-report. However, a more qualitative approach including ‘think-aloud’ interviews may have led to wordings different to those that were selected for use in our final questionnaire. Data collection through a survey sampling company tends to draw a narrow sample of people from similar ethnic and social backgrounds; although we had a good mix of educational levels, our sample was predominantly white. Weight and height measurements were self-reported, which may have led to over-estimation of height and under-reporting of weight (Gorber, Tremblay, Moher, & Gorber, 2007), potentially explaining why associations between BMI and appetitive traits were small. The cross-sectional nature of the study precludes any inferences about causation.

Although we established the factor structure, internal consistency, and test-retest reliability of the AEBQ, there was no comparison against behavioural measures of eating to assess validity. The CEBQ has been validated against several aspects of eating behaviour (including eating without hunger, caloric compensation, eating rate and energy intake at a meal) (Carnell & Wardle, 2007). Given the AEBQ is adapted from the CEBQ and has a similar factor structure there is no reason to believe its validity would differ, however this should be confirmed in future studies. Finally, as participants may have been aware that eating behaviours are related to weight, some individuals may have felt the need to respond to the AEBQ in a socially desirable way (Carnell and Wardle, 2008, Carnell et al., 2013).

In sum, the AEBQ, a self-report measure of appetitive traits in adults, is a reliable instrument, and provides a comprehensive, convenient, and easy-to-use measure of an adult’s appetite. The development of the AEBQ is an important step that permits large-scale research into key appetitive traits in adults, which are related to weight in infant and child populations. The relationships between appetitive traits and BMI in adulthood in this study were comparable to those observed in children, indicating that approach-related and avoidance-related appetitive traits are systematically (and oppositely) associated with BMI across the life-course. Future research should seek to replicate these findings in larger samples and using longitudinal designs, and to explore the potential for the AEBQ to inform weight control interventions.

The scoring system of the AEBQ can be downloaded from the following website: http://www.ucl.ac.uk/hbrc/resources/resources_eb.

## Figures and Tables

**Table:** 

**AEBQ**	**Item source**	**AEBQ item**	**CEBQ item**
H	New	I often feel so hungry that I have to eat something right away	
New	I often notice my stomach rumbling	
New	If I miss a meal I get irritable	
New	If my meals are delayed I get light-headed	
New	I often feel hungry	
FR	New	I often feel hungry when I am with someone who is eating	
New	When I see or smell food that I like, it makes me want to eat	
CEBQ (FR)	Given the choice, I would eat most of the time	Given the choice, my child would eat most of the time
NI	-	Even if my child is full up s/he finds room to eat his/her favourite food
NI	-	If given the chance, my child would always have food in his/her mouth
CEBQ (FR)	I am always thinking about food	My child is always asking for food
NI	-	If allowed to, my child would eat too much
EOE	CEBQ (EOE)	I eat more when I'm annoyed	My child eats more when annoyed
CEBQ (EOE)	I eat more when I'm worried	My child eats more when worried
New	I eat more when I'm upset	NI
CEBQ (EOE)	I eat more when I'm anxious	My child eats more when anxious
New	I eat more when I'm angry	NI
NI	-	My child eats more when s/he has nothing else to do
EF	CEBQ (EF)	I love food	My child loves food
CEBQ (EF)	I look forward to mealtimes	My child looks forward to mealtimes
CEBQ (EF)	I enjoy eating	My child enjoys eating
NI	-	My child is interested in food
SR	CEBQ (SR)	I often leave food on my plate at the end of a meal	My child leaves food on his/her plate at the end of a meal
CEBQ (SR)	I often get full before my meal is finished	My child gets full before his/her meal is finished
CEBQ (SR)	I get full up easily	My child gets full up easily
CEBQ (SR)	I cannot eat a meal if I have had a snack just before	My child cannot eat a meal if s/he has had a snack just before
NI	-	My child has a big appetite*
EUE	New	I eat less when I'm worried	
CEBQ (EUE)	I eat less when I'm angry	My child eats less when angry
CEBQ (EUE)	I eat less when I'm upset	My child eats less when upset
New	I eat less when I'm annoyed	
New	I eat less when I'm anxious	
NI	-	My child eats more when she is happy
NI	-	My child eats less when s/he is tired
FF	CEBQ (FF)	I refuse new foods at first	My child refuses new foods at first
NI	-	My child is difficult to please with meals
CEBQ (FF)	I often decide that I don’t like a food, before tasting it	My child decides that s/he doesn’t like a food, even without tasting it
CEBQ (FF)	I enjoy tasting new foods*	My child enjoys tasting new foods*
CEBQ (FF)	I am interested in tasting food I haven't tasted before*	My child is interested in tasting food s/he hasn’t tasted before*
CEBQ (FF)	I enjoy a wide variety of foods*	My child enjoys a wide variety of foods*
SE	CEBQ (SE)	I eat slowly	My child eats slowly
CEBQ (SE)	I am often last at finishing a meal	My child takes more than 30 minutes to finish a meal
CEBQ (SE)	I eat more and more slowly during the course of a meal	My child eats more and more slowly during the course of a meal
CEBQ (SE)	I often finish my meal (s) quickly*	My child finishes his/her meal quickly*

H, ‘hunger’; FR, ‘food responsiveness’; EOE, ‘emotional over-eating’; EF, ‘enjoyment of food’; SR, ‘satiety responsiveness’; EUE, ‘emotional under-eating’; FF, ‘food fussiness’; SE, ‘slowness in eating’.

Response options for the CEBQ: ‘never’, ‘rarely’, ‘sometimes’, ‘often’ and ‘always’.

Response options for the AEBQ: ‘strongly disagree’, ‘disagree’, ‘neither agree not disagree’, ‘agree’ and ‘strongly agree’.

*Indicates item should be reverse scored for calculating scale means or Chronbach’s alphas

**Table 1:** Descriptive statistics of adult samples used to carry out PCA (Sample 1) and CFA and re-test sample (Sample 2).

	Sample 1	Sample 2	
PCA (n = 708)	CFA (n = 954)	Re-test (n = 93)
n (%)	n (%)	n (%)
**Age**			
18 to 29	301 (42.5%)	166 (17.4%)	9 (9.7%)
30 to 59	300 (42.4%)	654 (68.6%)	59 (63.4%)
60+	107 (15.1%)	134 (14.0%)	25 (26.9%)
**Gender**			
M	336 (47.5%)	407 (42.7%)	19 (20.4%)
F	372 (52.5%)	547 (57.3%)	74 (79.6%)
**BMI**^a^			**n** = **90**

Underweight	30 (4.4%)	25 (2.7%)	2 (2.2%)
Normal weight	328 (48.7%)	380 (39.8%)	40 (44.4%)
Overweight	173 (25.6%)	278 (29.1%)	25 (27.8%)
Obese	143 (21.2%)	257 (26.9%)	23 (24.7%)
**Ethnicity**			
White	635 (90.3%)	863 (90.5%)	91 (97.8%)
Non-white	68 (9.7%)	91 (9.5%)	2 (2.2%)
**Education**			
Finished primary/secondary school or O level/GCSE^b^	179 (25.6%)	243 (25.5%)	28 (30.1%)
A levels or technical or trade certificate or diploma	242 (34.6%)	359 (37.6%)	29 (31.2%)
University	279 (39.9%)	352 (36.9%)	36 (38.7%)

a Participants who reported a BMI <14 or >50 were excluded as these values were felt to be unrealistic.

b General Certificate of Secondary Education.

**Table 2:**
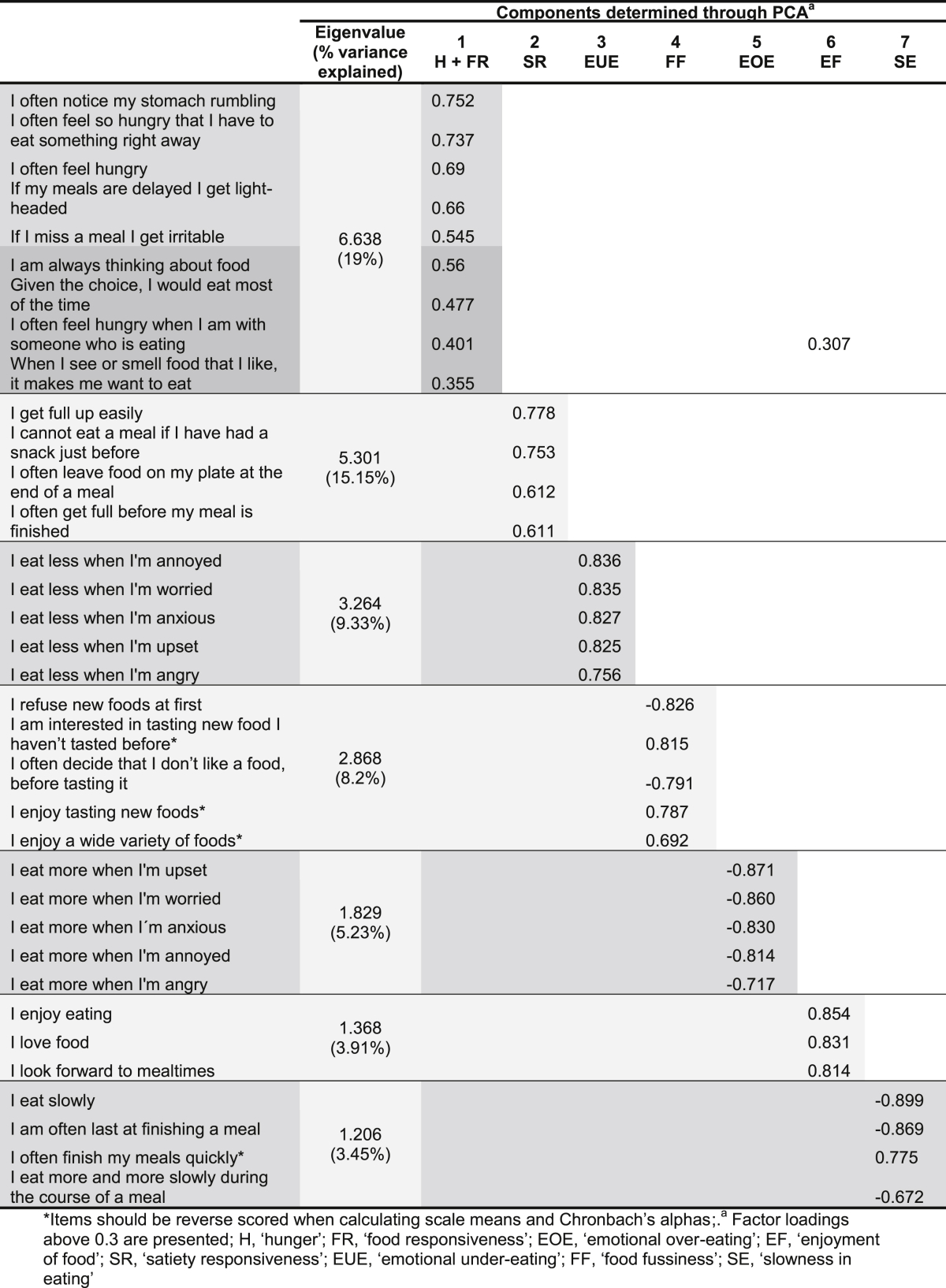
Factor loadings of a 35 item AEBQ (Sample 1, n = 708).

**Table 3:** Model fit indices of two CFA Models of the AEBQ (Sample 2, n = 954).

Model	Items	Factors	Exogenous variables	NFI	CFI	RMSEA	χ^2^	df	AIC	BIC
Model 1	35	7 (H + FR on a single factor)	42	0.871	0.896	0.061	2431.345	539	2613.345	3055.665
Model 2	35	8 (H + FR as separate factors)	43	0.880	0.905	0.058	2254.657	532	2450.657	2927.002

H, ‘Hunger’; FR, ‘Food Responsiveness’; NFI, ‘Normed Fixed Index’; CFI, ‘Comparative Fixed Index’; RMSEA, ‘Root Mean Square Error of Approximation’; χ^2^, ‘Chi-square’; df, ‘degrees of freedom’; AIC, ‘Akaike’s Information Criteria’; BIC, ‘Bayesian Information Criterion’.

**Table 4:** Internal and test-retest reliability for the AEBQ (Sample 2, n = 954).

AEBQ scales	Internal reliability (Cronbach’s alphas) (n = 954)	Test re-test reliability (Intraclass correlations, 95% confidence intervals) (n = 93)
Hunger^a^	0.751	0.821 (0.730–0.881)
Food responsiveness^a^	0.753	0.871 (0.805–0.914)
Emotional over-eating^a^	0.904	0.732 (0.596–0.823)
Enjoyment of food^a^	0.859	0.860 (0.789–0.907)
Satiety responsiveness^b^	0.753	0.865 (0.797–0.911)
Emotional under-eating^b^	0.896	0.772 (0.656–0.849)
Food fussiness^b^	0.877	0.907 (0.860–0.939)
Slowness in eating^b^	0.884	0.910 (0.864–0.940)

a Food approach scales.

b Food avoidance scales.

**Table 5:** Correlations between appetitive traits (Sample 2; n = 954).

		Food approach scales			Food avoidance scales			
FR	EOE	EF^a^	SR	EUE	FF	SE
Food approach scales	H	0.621^c^	0.362^c^	0.344^c^	−0.043	0.118^c^	−0.029	−0.048
FR	–	0.439^c^	0.551^c^	−0.233^c^	−0.033	−0.102^b^	−0.207^c^
EOE		–	0.194^c^	−0.139^c^	−0.321^c^	0.085^c^	−0.136^c^
EF^a^			–	−0.283^c^	−0.103^c^	−0.356^c^	−0.197^c^
Food avoidance scales	SR				–	0.300^c^	0.200^c^	0.465^c^
EUE					–	0.025	0.206^c^
FF						–	0.063

H, ‘hunger’; FR, ‘food responsiveness’; EOE, ‘emotional over-eating’; EF, ‘enjoyment of food’; SR, ‘satiety responsiveness’; EUE, ‘emotional under-eating’; FF, ‘food fussiness’; SE, ‘slowness in eating’.

a Pearson’s correlation was used for normally distributed mean scores, except for ‘enjoyment of food’ where Spearman’s rho was used.

b Correlation is significant at the 0.05 level (2-tailed).

c Correlation is significant at the 0.01 level (2-tailed).

**Table 6:** Correlations between AEBQ and BMI (Sample 2) (n = 940).

	Food approach scales				Food avoidance scales			
Hunger	Food responsive-ness	Emotional over-eating	Enjoyment of food	Satiety responsive-ness	Emotional under-eating	Food fussiness	Slowness in eating
BMI	−0.028	0.071^a^	0.259^b^	0.067^a^	−0.127^b^	−0.202^b^	0.033	−0.108^b^

a Correlation is significant at the 0.05 level (2-tailed).

b Correlation is significant at the 0.01 level (2-tailed).

## References

[bib1] Ashcroft J., Semmler C., Carnell S., van Jaarsveld C.H.M., Wardle J. (2008). Continuity and stability of eating behaviour traits in children. European Journal of Clinical Nutrition.

[bib2] Banna J.C., Vera Becerra L.E., Kaiser L.L., Townsend M.S. (2010). Using qualitative methods to improve questionnaires for Spanish speakers: assessing face validity of a food behavior checklist. Journal of the American Dietetic Association.

[bib3] Carnell S., Benson L., Pryor K., Driggin E. (2013). Appetitive traits from infancy to adolescence: using behavioral and neural measures to investigate obesity risk. Physiology & Behavior.

[bib4] Carnell S., Wardle J. (2007). Measuring behavioural susceptibility to obesity: validation of the child eating behaviour questionnaire. Appetite.

[bib5] Carnell S., Wardle J. (2008). Appetite and adiposity in children: evidence for a behavioral susceptibility theory of obesity. The American Journal of Clinical Nutrition.

[bib6] Croker H., Cooke L., Wardle J. (2011). Appetitive behaviours of children attending obesity treatment. Appetite.

[bib7] Dugard P., Todman J., Staines H. (2010). Factor analysis. Approaching multivariate analysis.

[bib8] Field A. (2013). Discovering statistics using SPSS.

[bib9] Flegal K.M., Kit B.K., Orpana H. (2013). Association of all-cause mortality with overweight and obesity using standard Body Mass index categories. a systematic review. American Medical Association.

[bib10] French S.A., Epstein L.H., Jeffery R.W., Blundell J.E., Wardle J. (2012). Eating behavior dimensions. Associations with energy intake and body weight. A review. Appetite.

[bib11] Fuemmeler B.F., Lovelady C.A., Zucker N.L., Ostbye T. (2013). Parental obesity moderates the relationship between childhood appetitive traits and weight. Obesity.

[bib12] Gorber C.S., Tremblay M., Moher D., Gorber B. (2007). A comparison of direct vs. self-report measures for assessing height, weight and body mass index: a systematic review. Obesity Reviews.

[bib13] Hu L., Bentler P.M. (1999). Cutoff criteria for fit indexes in covariance structure analysis: conventional criteria versus new alternatives. Structural Equation Modeling.

[bib14] Karlsson J., Persson L.O., Sjöström L., Sullivan M. (2000). Psychometric properties and factor structure of the Three-Factor Eating Questionnaire (TFEQ) in obese men and women. Results from the Swedish Obese Subjects (SOS) study. International Journal of Obesity.

[bib15] Kauer J., Pelchat M.L., Rozin P., Zickgraf H.F. (2015). Adult picky eating. Phenomenology, taste sensitivity, and psychological correlates. Appetite.

[bib16] Llewellyn C.H., van Jaarsveld C.H.M., Johnson L., Carnell S., Wardle J. (2011). Development and factor structure of the Baby Eating Behaviour Questionnaire in the Gemini birth cohort. Appetite.

[bib17] Llewellyn C.H., van Jaarsveld C.H.M., Plomin R., Fisher A., Wardle J. (2012). Inherited behavioral susceptibility to adiposity in infancy: a multivariate genetic analysis of appetite and weight in the Gemini birth cohort. American Journal of Clinical Nutrition.

[bib18] Llewellyn C.H., Wardle J. (2015). Behavioral susceptibility to obesity: gene–environment interplay in the development of weight. Physiology & Behavior.

[bib19] Loh D.A., Moy F.M., Zaharan N.L., Mohamed Z. (2013). Eating behaviour among multi-ethnic adolescents in a middle-income country as measured by the self-reported Children’s Eating Behaviour Questionnaire. PLoS ONE.

[bib20] Mallan K.M., Liu W.H., Mehta R.J., Daniels L.A., Magarey A., Battistutta D. (2013). Maternal report of young children’s eating styles. Validation of the Children’s Eating Behaviour Questionnaire in three ethnically diverse Australian samples. Appetite.

[bib21] McGraw K.O., Fleiss J.L. (1996). Forming inferences about some intraclass correlation coefficients. Psychological Methods.

[bib22] Meyer J.E., Pudel V.E. (1972). Experimental studies in obese and normal weight subjects. Journal of Psychosomatic Research.

[bib23] Ng M., Fleming T., Robinson M., Thomson B., Graetz N., Margono C. (2014). Global, regional, and national prevalence of overweight and obesity in children and adults during 1980-2013: a systematic analysis for the Global Burden of Disease Study 2013. Lancet.

[bib24] Santos J.L., Ho-Urriola J.A., González A., Smalley S.V., Domínguez-Vásquez P., Cataldo R. (2011). Association between eating behavior scores and obesity in Chilean children. Nutrition Journal.

[bib25] Schachter S. (1968). Obesity and eating. Internal and external cues differentially affect the eating behaviour of obese and normal subjects. Science.

[bib26] Schachter S., Gross L.P. (1968). Manipulated time and eating behavior. Journal of Personality and Social Psychology.

[bib27] Sleddens E.F., Kremers S.P., Thijs C. (2008). The children’s eating behaviour questionnaire: factorial validity and association with Body Mass Index in Dutch children aged 6–7. The International Journal of Behavioral Nutrition and Physical Activity.

[bib28] Spence J.C., Carson V., Casey L., Boule N. (2011). Examining behavioural susceptibility to obesity among Canadian pre-school children: the role of eating behaviours. International Journal of Pediatric Obesity: IJPO: An Official Journal of the International Association for the Study of Obesity.

[bib30] Stunkard A.J., Fox S. (1971). The relationship of gastric motility and hunger. A summary of the evidence. Psychosomatic Medicine.

[bib31] Stunkard A.J., Messick S. (1985). The Three-Factor Eating Questionnaire to measure dietary restraint, disinhibition and hunger. Journal of Psychosomatic Research.

[bib32] Svensson V., Lundborg L., Cao Y.T., Nowicka P., Marcus C., Sobko T. (2011). Obesity related eating behaviour patterns in Swedish preschool children and association with age, gender, relative weight and parental weight–factorial validation of the Children’s Eating Behaviour Questionnaire. The International Journal of Behavioral Nutrition and Physical Activity.

[bib33] Sweetman C., Wardle J., Cooke L. (2008). Soft drinks and “desire to drink” in preschoolers. The International Journal of Behavioral Nutrition and Physical Activity.

[bib34] Van Jaarsveld C.H.M., Llewellyn C.H., Johnson L., Wardle J. (2011). Prospective associations between appetitive traits and weight gain in infancy. The American Journal of Clinical Nutrition.

[bib35] Van Strein T., Frijters J.E.R., Bergers G.P.A., Defares P.B. (1986). The Dutch Eating Behavior Questionnaire (DEBQ) for assessment of restrained, emotional, and external eating behavior. International Journal of Eating Disorders.

[bib37] Viana V., Sinde S., Saxton J.C. (2008). Children’s Eating Behaviour Questionnaire: associations with BMI in Portuguese children. The British Journal of Nutrition.

[bib38] Wardle J. (1987). Hunger and satiety: a multidimensional assessment of responses to caloric loads. Physiology & Behavior.

[bib39] Wardle J., Guthrie C.A., Sanderson S., Rapoport L. (2001). Development of the Children’s Eating Behaviour Questionnaire. Journal of Child Psychology and Psychiatry.

[bib40] Wardle, J., Liao, L.-M., Rapoport, L., Hillsdon, M., Croker, H., & Edwards, C. (2013). Shape-Up. A self-help guide to managing your weight. (J. Wardle, P. Chadwick, A. Chipperfield, H. Croker, N. Gokool, L.-M. Liao, et al., Eds.). London, UK: Weight Concern.

[bib41] Webber L., Hill C., Saxton J., Van Jaarsveld C.H.M., Wardle J. (2009). Eating behaviour and weight in children. International Journal of Obesity.

